# *Tetrapleura tetraptera* spice attenuates high-carbohydrate, high-fat diet-induced obese and type 2 diabetic rats with metabolic syndrome features

**DOI:** 10.1186/s12944-015-0051-0

**Published:** 2015-05-24

**Authors:** Dieudonne Kuate, Anne Pascale Nouemsi Kengne, Cabral Prosper Nya Biapa, Boris Gabin Kingue Azantsa, Wan Abdul Manan Bin Wan Muda

**Affiliations:** Program in Nutrition, School of Health Sciences Universiti Sains Malaysia, Kubang Kerian, Kelantan 16150 Malaysia; Department of biochemistry, Faculty of science, University of Dschang, PO Box 67, Dschang, Cameroon; Department of Biochemistry and Molecular Biology, Faculty of science, University of Buea, Buea, Cameroon

**Keywords:** Obesity, Type 2 diabetes, High-carbohydrate high-fat diet, Metabolic syndrome, Inflammation, *Tetrapleura tetraptera*

## Abstract

**Background:**

*Tetrapleura tetraptera*, a seasoning and nutritive spice is also used in western African folk medicine in the management of wide variety of diseases including diabetes, inflammation and hypertension. Flavonoids and saponins are some abundant secondary metabolic constituents in the fruits of this plant. This study aimed at evaluating the potential therapeutic action of the polyphenol-rich hydroethanolic extract (HET) of this fruit in experimentally induced obese and type 2 diabetic rats (T2DM) with characteristic metabolic syndrome (MetS).

**Methods:**

MetS was induced in rats by high-carbohydrate, high-fat diet and administration of low-dose streptozotocin. Then different oral doses of HET (200 and 400 mg/kg) were administered to T2DM rats for 28 days. A standard antidiabetic drug, metformin (300 mg/kg), was used for comparison. The body weight, systolic blood pressure, oxidative stress and metabolic parameters were then assessed to evaluate the effect of HET on MetS.

**Results:**

HET reduced weight gain, fasting blood glucose and plasma insulin levels as well as homeostasis model assessment of insulin resistance (HOMA-IR) and alleviated obesity and T2DM associated oxidative stress and hypertension in rats. Moreover, a significantly hypolipidemic property and an attenuation of liver injury and tissue steatosis was observed after HET administration. HET further demonstrated its anti-inflammation effect via down regulation of tumor necrosis factor-alpha (TNF-α), interleukin-6 (IL-6), C-reactive protein (CRP), leptin and an increase in adiponectin. The HET exhibited dose-dependent effects which were comparable to that of metformin.

**Conclusions:**

The present study thereby demonstrates the anti-insulin resistance, antilipidemic, anti-obesity, hypotensive and anti-inflammatory properties of HET; hence it has the potential to be further developed for the management of MetS such as obesity, T2DM and hypertension.

## Introduction

Obesity, diabetes and hypertension are increasingly common human health conditions which are referred to as metabolic syndrome. Metablic syndrome is defined as the clustering of risk factors for cardiovascular disease and type 2 diabetes increases cardiovascular mortality. Diabetes mellitus, is the most common metabolic disorder characterized by impaired homeostasis of carbohydrate, protein, and fat metabolism which ultimately lead to persistent elevated blood glucose levels. The most recent estimates of International Diabetes Federation (IDF) indicate that 8.3 % of adults – 382 million people – have diabetes in 2013, and the number of people with the disease is set to rise beyond 592 million by 2035, if current demographic patterns continue [1]. The underlying cause of diabetes is either decrease in the synthesis/secretion of insulin (type 1 diabetes mellitus (T1DM)) or by insulin resistance, followed later by death of pancreas’ β-cells (T2DM). T2DM which constitutes the vast majority of diabetes is a heterogeneous disorder characterized by impairment of insulin sensitivity, followed by the inability of pancreatic β cells to compensate for insulin resistance (pancreatic beta cell dysfunction) [2].

Obesity results from rapid urbanization, western-style high-fat high-carbohydrate diet and a sedentary lifestyle. Visceral obesity is the major risk factor contributing to the development of insulin resistance. In addition, oxidative stress resulting from persistent hyperglycemia, contributes to the vicious cycle of insulin resistance and low-grade inflammation, characterized by changes in biochemical markers of inflammation and the activation of some proinflammatory signaling pathways [2]. All these biochemical changes are often concomitant with excess lipid accumulation in adipose tissues [3]. Infiltrated immune cells enhance the low-grade inflammation in adipose tissues, and the increased inflammatory cytokines subsequently stimulate insulin resistance and inflammation in other organs such as the skeletal muscle and liver [4]. Hyperlipidemia, hyperglycemia, inflammation and oxidative stress are therefore playing an important role in the pathogenesis of T2DM, leading to the development of microvascular and macrovascular complications of diabetes, which further contribute to the morbidities and mortalities related to T2DM and cardiovascular diseases [5]. Thus, besides controlling weight and hyperglycemia, efficient amelioration of dyslipidemia, inflammation and oxidative stress are important factors in clinical treatment of obesity and T2DM. The drugs currently used as antidiabetic medicines include synthetic agents such as biguanides, thiazolidinediones, insulin sensitizers and insulin and these come with considerable side effects, such as hypoglycemia, drug resistance, dropsy and weight gain as well as limited hypolipidemic, anti-inflammatory and antioxidant activities [6]. Because they are more effective, safer and affordable, a wide variety of bioactive components or phytochemicals in foods and functional foods have become popular and have been considered as complementary or alternative therapies for the management and/or treatment of chronic diseases [2, 7, 8]. Moreover, it is reported that in addition to standard prescribed drugs, a host of complementary and alternative exotic fruits, herbs, spices and dietary supplements are widely used in the prevention/treatment of obesity, diabetes and metabolic syndrome [9–12]. The purified bioactive compounds and/or whole extract from medicinal foods have various targets to ameliorate these health ailments, related complications, hence their burden on the healthcare.

A wide range of spices is commonly used in the Cameroon, many of which are native to the country. Apart from their flavoring characteristics, a number of these spices are highly valued locally because of their putative medicinal properties [13, 11]. *Tetrapleura tetraptera* (Schumach. And Thonn) Taub, is a leguminous multipurpose tree (Mimosoideae) indigenous to tropical Africa. It is an exotic fruit tree known in Western Nigeria as Aridan. It is commonly used to prepare several traditional dishes as popular seasoning spice in western African countries, including Ghana, Nigeria and Cameroon while Ghanaians use the fruit as multivitamins [14]. Compared with other commonly used spices, it is a rich source of phytochemicals which contribute to its documented biological and pharmacological activities, including cardiovascular, anti-inflammatory, hypoglycaemic, hypotensive, neuromuscular, anti-convulsant, molluscicidal, trypanocidal, hirudinicidal, anti-ulcerative, ectoxicity, anti-microbial, emulsifying property, birth control, food value and the control of intestinal parasites [15–17]. The nutrients and anti-nutrients content of *Tetrapleura tetraptera* fruits have also been reported [13, 17, 18]. The phytochemical composition in the fruits of *Tetrapleura tetraptera* includes polyphenols (tannins, flavonoids), saponins, phytate, triterpenoid, coumarinic (scopoletin) and phenolic (caffeic acid, cinnamic acids) compounds, a triterpene glycoside (aridanin) which have been found as the active ingredients [7, 17, 19–21]. In a preliminary study, among water, ethanol and hydroethanolic extracts, the hydroethanolic extract showed the highest significant blood glucose lowering effect as well as the highest polyphenolic content. In this study, we investigated the dose-dependent effect of a hydroethanolic extract of *Tetrapleura tetraptera* (HET) on the obese and T2DM rats model (high-carbohydrate high-fat diet and low-dose streptozotocin (STZ) induced diabetic rats) with characteristics of insulin resistance, obesity, inflammation, hyperlipidemia, oxidative stress and hypertension.

## Materials and methods

### Preparation of the polyphenol-rich hydroethanolic extract of *Tetrapleura tetraptera*

The dried fruits of TT were bought from the local market in Yaoundé, Cameroon and identified at the national herbarium. Pulps were isolated, ground and extracted using a mixture of ethanol and water (50:50). Briefly, 5 kg of pulps were shade dried and powdered, then extracted with 50 % ethanol for 24 h. After filtration and evaporation of the solvent, the extract was obtained (450 g).

### Experimental animals

Adult male albino rats of Sprague–Dawley (SD) ((180–210 g), age 6 weeks) strain were used for the experiment. The animals were housed in polypropylene cages and maintained under controlled conditions of temperature (24 () 2 °C) in 12-h light/12-h dark cycles, 50 % humidity. This study was approved by Institutional Review Board for animal care and the study was conducted in accordance with the “Guide for the Care and Use of Laboratory Animals”

### Experimental design and induction of high-carbohydrate high-fat obese and type 2 diabetic rats

Obesity and T2DM was induced according to the modified method of Peng et al. [22]. Briefly, male SD rats were acclimatized and fed a basic chow diet consisting of 7 % fat during the first week before experimentation. All animals had free access to water and food. The rats were divided into the groups of six as follows: normo lipidic control diet (NCD), high-carbohydrate high-fat diet (HCHFD), HCHFD+ HET (HCHFD treated with 200 mg/kg of HET) (HCHFD200), HCHFD + STZ (DBC), HCHFD + STZ + Metformin (300 mg/kg) (DBMET), HCHFD + STZ + HET (low: 200 mg/kg HET) (DB200), and HCHFD+ STZ + HET (high: 400 mg/kg HET) (DB400). After 7 weeks of high fat high carbohydrate diet feeding, when the average body weight reached 410()15 g, the high-carbohydrate high-fat diet fed rats were divided into HCHFD and HCHFD + STZ groups. The latter were injected intraperitoneally with a single dose (30 mg/kg bw) of freshly prepared streptozotocin (STZ) in citrate buffer (0.1 M, pH 4.5). The other groups received only the same amount of 0.1 M citric acid buffer (pH 4.5). One week later, rats with a fasting blood glucose level ≥ 11.1 mmol/L were considered as T2DM rats and used for the experiment. Rats were then tube-fed for 4 weeks with or without different doses of HET (via oral gavage) or metformin (at a dose of 300 mg/kg, which is in-between those of the extract), a standard antidiabetic drug used as a positive control. The same amount of drinking water was used to serve as the blank. The body weight was measured weekly throughout the study. Food and water intakes were measured daily for all rats.

During the experiment, rats were fed two control diets: a normo lipidic control diet, prepared in accordance with the American Institute of Nutrition [23] AIN-93G, with a protein concentration of 12 % and a high-carbohydrate high-fat control diet AIN‐93G-modified, with 12 % (g/g) protein, 40 % carbohydrate, 35 % (g/g) fat, (4 % (g/g) vegetable oil (corn) and 31 % (g/g) of animal origin (beef tallow)) and a drinking solution of 30 % carbohydrate (sucrose).

The composition of the high-carbohydrate high-fat diet was as follows (g/100 g food): corn starch, 25;maltodextrin, 8,3; corn oil, 4; beef tallow,31; casein (78 % protein), 15.4; sucrose, 6.3; cellulose, 5.0; mineral premix, 3.5; choline bitartrate, 0,2; and vitamin premix, 1; L-cystine 0.3; In addition to this 30 % sucrose solution was provided as the drinking fluid to HCHFD rats. The normolipidic control diet contained corn starch, 47; maltodextrin, 14.2; sucrose, 6.3; corn oil, 7; no beef tallow; normal drinking water, and the remainder unchanged. Mineral and vitamin mixtures, as described in AIN-76 were purchased from Harlan Teklad (Madison, USA). Both STZ injected and non-injected animals continued on their original diet for the duration of the study. The vehicle control group received normal saline. At days 0 and 28 of treatment, blood samples were collected by the retro-orbital sinus puncture under chloroform anesthesia. The fasting blood glucose levels were determined using a glucometer (ONETOUCH, Ultra, Lifescan, USA). At day 21 oral glucose tolerance test was performed.

### Biochemical analysis

After 28 days of treatment and before sacrifice for organ collection, the animals were fasted overnight and weighted, blood samples were then collected by retro-orbital sinus puncture using capillary under chloroform anesthesia. Two different tubes with and without anticoagulant (heparin) were used for blood collection in each rat to obtain serum and plasma. Then, serum and plasma were prepared by centrifuging the blood samples at 4000 rpm for 15 min. Plasma total cholesterol (TC), triglycerides (TG), and high-density lipoprotein-cholesterol (HDL-C) were estimated enzymatically using standard kits (Accurex Biomedical Pvt. Ltd., Thane, India), whereas low-density lipoprotein-cholesterol (LDL-C) were calculated based on Friedewald’s equation. LDL-C (mg/dL) = TC- (HDL-C)- (TG/5). Serum activity of liver function enzymes: alanine transaminase (ALT/GPT), aspartate transaminase (AST/GOT), and the concentrations of total protein, urea and uric acid were estimated by using kits specific for the each test using a semi-autoanalyzer (Photometer 5010 V5+, Germany).

The plasma levels of malondialdehyde (MDA), glutathione (GSH), superoxide dismutase (SOD), free fatty acids (FFA), were determined by using the corresponding assay kits according to manufacturer’s guidelines. The concentrations of the serum interleukin-6 (IL-6), tumor necrosis factor-α (TNF-α), C-reactive protein (CRP), leptin, plasma insulin levels and adiponectin were measured using the enzyme-linked immunosorbent assay (ELISA) method using commercially available kits according to the manufacturer’s instructions (R&D Systems Inc., Minneapolis, MN, USA; Millipore Co., Billerica, Massachusetts, USA). Glycosylated hemoglobin (HbA_1_C) levels were measured using commercial NycoCard HbA1C Test kits (Axis-Shield Company, Oslo, Norway) and a NycoCard HbA_1_C Reader II. The Advanced Glycation End Products (AGE) were determined using the OxiSelect Advanced Glycation End Product ELISA Kit (Cell Biolabs, CA) according to the manufacturer’s instructions. All samples and standards were evaluated in triplicates to ensure accuracy of results.

### Measurement of TC, TG and FFA contents in rat liver and skeletal muscle

At the end of the experiment the animals were euthanized under chloroform anaesthesia and livers and skeletal muscles (the quadriceps from the left hind limb of the animals) were quickly excised off, immediately rinsed in ice cold saline and stored in liquid nitrogen tank. Portions of these tissues (100 mg) were washed with saline and homogenized in 2 ml chloroform/methanol (2:1) for lipid extraction. After homogenization, lipids were further extracted by rocking samples for 1 h at room temperature, followed by centrifugation at 5000 rpm for 10 min. The liquid phase was washed with 0.2 volume of 0.9 % saline. The mixture was centrifuged again at 2000 rpm for 5 min to separate the two phases. The lower phase containing lipids was evaporated and lipids were dissolved in 0.5 ml isopropanol containing 10 % Triton X-100 for TG and TC measurements as described above.

### Determination of insulin resistance

The oral glucose tolerance test (OGTT) was performed on day 21 on fasted rats. During this fasting period, fructose-supplemented drinking water in HSHF groups was replaced with normal drinking water. Blood glucose was determined at t = 0 through a small incision in the caudal vein, followed by intra-peritoneal injection of glucose solution 25 % (2 g/kg), 30 min after the administration of *T. tetraptera*. The blood glucose was again measured at 30, 60 and 120 min in order to determine the glucose level increment. At days 0 (baseline) and 28, the homeostasis model assessment of insulin resistance (HOMA-IR) and HOMA-β scores were calculated according to the method of Mathews et al. [24] using fasting plasma insulin (FI) and fasting blood glucose (FBG) concentrations at the baseline and the end of the experimental period according to the following formula:

$$ \mathrm{HOMA}\hbox{-} \mathrm{I}\mathrm{R}=\left[\mathrm{F}\mathrm{I}\left(\upmu \mathrm{I}\mathrm{U}/\mathrm{mL}\right)\times \mathrm{F}\mathrm{B}\mathrm{G}\;\left(\mathrm{mmol}/\mathrm{L}\right)\kern0.5em \right]\;/\;22.5 $$

$$ \mathrm{HOMA}\hbox{-}\;\upbeta =\left[20\times \mathrm{F}\mathrm{I}\kern0.1em \left(\upmu \mathrm{I}\mathrm{U}/\mathrm{mL}\right)\right]\kern0.22em /\;\left[\mathrm{F}\mathrm{B}\mathrm{G}\kern0.1em \left(\mathrm{mmol}/\mathrm{L}\right)-3.5\right] $$

Conversion factor: Insulin (1μIU/ml = 0.0417 ng/mL = 7.175pmol/L) and blood glucose (1 mmol/L = 18 mg / dl).

### Systolic blood pressure measurements

The systolic blood pressure (SBP) of the tail artery was measured on the 28^th^ day under anesthesia, by non-invasive blood pressure system MODEL BP-6, Diagnostic & Research Instruments Co. Ltd., Taoyuan, Taiwan). The measurements for SBP were recorded in quadruplicates for each rat and the average systolic blood pressure was calculated.

### Statistical analysis

All results were expressed as median (range) and each group consisted of 6 rats. Groups were compared by Kruskal-Wallis test. Differences between two groups were identified by Mann–Whitney test. P < 0.05 was considered statistically significant. All the statistical analyses were carried out using the Statistical Package for Social Sciences version 20 (SPSS Inc., Chicago, USA).

## Results

### HET possessed a hypoglycemic ability

As shown in Table 1, HCHF diet significantly affects the blood glucose level, though there was no frank hyperglycemia such as in diabetic group (HCHFD + STZ = DBC) where glycaemia increased to about 3 fold compared with the normal control (NCD) group. Two hundred mg/kg dose of HET significantly decreased the high glucose level in obese rats whereas both 200 and 400 mg/kg doses significantly reduced the diabetic high glucose by about 50 % and 65 %, respectively. This indicates that HET possessed a hypoglycemic effect in rats with characteristics of metabolic syndrome. The effect of the higher dose was even greater than that of metformin (300 mg/kg).

**Table 1:** Fasting blood glucose and fasting insulin of control and experimental rats at baseline and after 28 days of treatment with *Tetrapleura tetraptera* hydroethanolic extract and metformin

Groups	Days	FBG (mmol/L)	Plasma insulin (μIU/mL)
NCD	0	5.28(4.61–5.94)^bc^	17.91(16.69–19.75)^bc^
	28	5.39(4.83–5.55)^bc^	17.88(17.12–19.34)^b^
	T28-T0	−0.23(−0.61–0.94)	−0.06(−0.78–0.43)
HCHFD	0	6.97(6.16–7.38)^abc^	40.3(38.95–45.91)^a^
	28	7.03(6.11–7.44)^abc^	47.35(44.15–49.13) ^Sac^
	T28-T0	0.05(−0.05–0.11)	5.95(2.43–9.37)
HCHFD200	0	6.72(6.27–7.38)^abc^	39.79(37.23–45.32)^a^
	28	5.44(4.66–6.16)^Sbc^	22.78(20.39–25.20)^Sb^
	T28-T0	−1.48(−2.11_−0.72)	−17.4(−22.3_−13.03)
DBC	0	17.11(12.27–19)^a^	36.25(34.12–39.23)^a^
	28	16.42(12.22–18.94)^a^	35.4(34.15–38.87)^a^
	T28-T0	−0.31(−1.33–0.06)^a^	−0.26(−1.2–0.04)
DB200	0	16.55(12.44–18.33)^Sabc^	37(35.48–38.26)^a^
	28	7.97(6.88–9.4)^Sabc^	24.74(22.31–27.01)^Sabc^
	T28-T0	−8(−9.39_−5.56)	−11.75(−14.91_−9.76)
DB400	0	16.92(13.16–18.45)^a^	36.15(35.11–39.36)^a^
	28	6.14(5.88_−7.05)^Sbc^	18.78(16.32–19.88)^Sbc^
	T28-T0	−10.31(−12.57_−7.05)	−17.36(−23.04_−15.57)
DBMET	0	16.61(14.44–18.11)^a^	36.33(35.14–39.15)^a^
	28	6.94(5.77–7.77)^Sbc^	20.62(19.22–23.15)^Sbc^
	T28-T0	−9.59(−11_−6.83)	−16.15(−18.97−13)

^S^significant compared with T0 (p < 0.05). ^a^significant relative to normal control on the same treatment day(p < 0.05). ^b^significant compared with HCHFD on the same treatment day. ^c^significant compared with diabetic control on the same treatment day (p < 0.05). (n = 6)

### HET reversed hyperinsulinemia accompanied with obesity and type 2 diabetes status

**Fig. 1:**
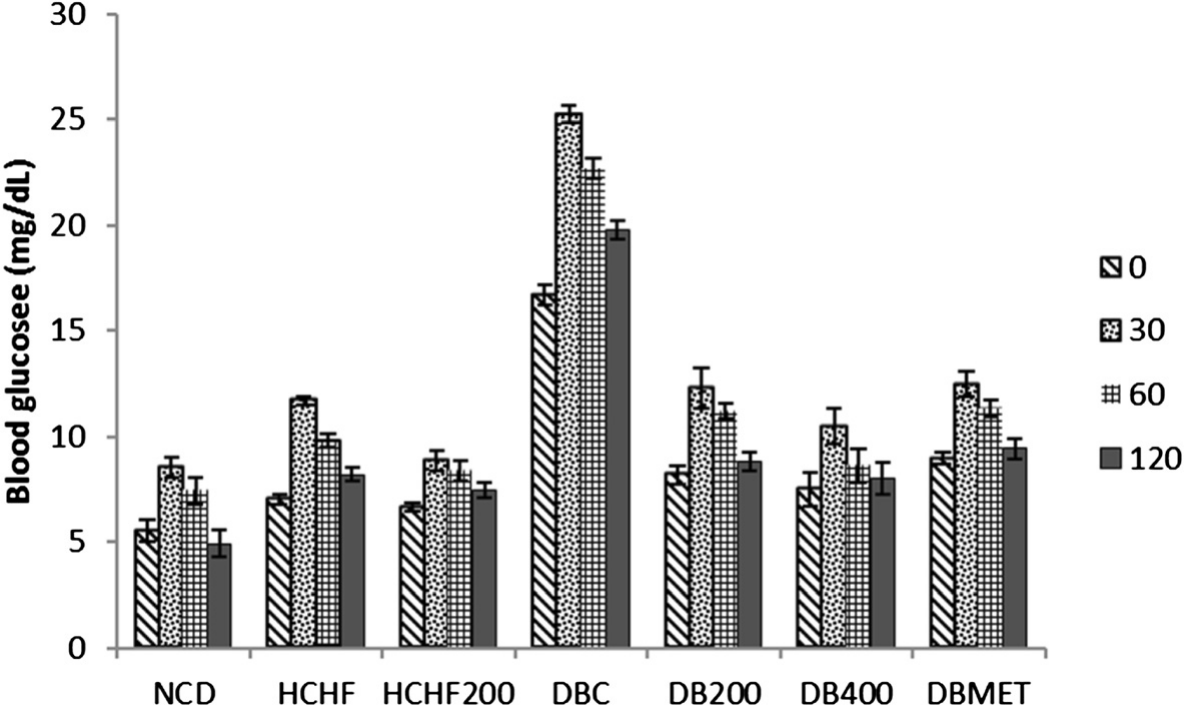
Effect of HET on OGTT After 21-day HET treatment, rats were fasted overnight followed by intra-peritoneal injection of glucose (2 g/kg). Their blood glucose levels were then measured at 0, 30, 60 and 120 min after glucose administration

Plasma insulin levels were assessed to investigate whether hyperglycemia status was accompanied with hyperinsulinemia, the prominent feature of type 2 diabetes. The HCHFD rats had reduced insulin sensitivity (Fig. 1) thus significant insulin resistance, but T2DM only occurred after the low-dose STZ injection. In this model, we observed that HCHF diet seemed to elevate the plasma insulin level almost by 2.5 fold whereas this increase was slightly reduced in HCHFD + STZ though the insulin level was still significantly higher by about 2-fold as compared with the NCD. We demonstrated that HET lowered the insulin level in a dose-dependent manner (Table 1). At a dose of 200 mg/kg, HET was able to ameliorate the obesity and type 2 diabetes induced hyperinsulinemia in rats, indicating its potential anti-insulin-resistance property.

**Fig. 2:**
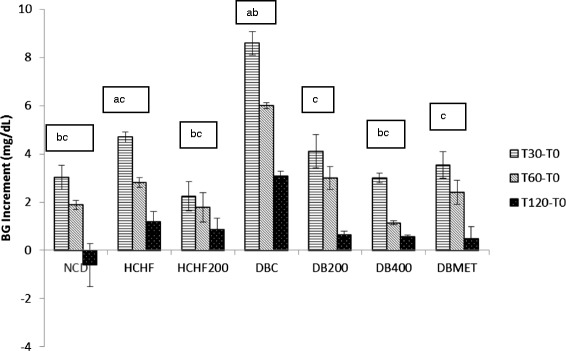
Effect of HET on OGTT. Glucose variation (increment) at different times values at 30,60 and 120 min minus that of baseline. ^a^significant relative to normal control (p < 0.05). ^b^significant compared with HCHFD (p < 0.05). ^c^significant compared with diabetic control (p < 0.05). (n = 6)

The HOMA-IR score was significantly higher in the HCHFD and DBC groups compared to the NC whereas the HOMA- β (pancreatic β -cell function) score was significantly lower in the DBC relative to NCD and HCHFD groups (Table 2). There was no significant difference in HOMA- β score between NCD and HCHFD groups indicating that the pancreatic β -cell function was not affected in HCHFD groups despite the high plasma insulin status. Likewise, HET concomitantly reduced the HOMA-IR score in HCHFD200, DB200 and DB400 groups and increased the HOMA- β in diabetic treated groups. It is well known that OGTT is a sensitive assessment of the early abnormalities in glucose regulation than fasting blood glucose or glycosylated hemoglobin. HCHF diet fed rats as well as diabetic rats showed an impaired glucose tolerance when compared with normal control group (Figs. 1 and 2), as indicated by the significant difference in blood glucose increment after 30 min. In our study, oral administration of both HET and metformin to HCHFD and diabetic rats showed a significant reduction in peak blood glucose level at 30 min in treated rats during OGTT (p < 0.05). HET (400 mg/kg) had more pronounced effect than the standard drug (300 mg/Kg).

**Table 2:** HOMA-IR and HOMA- β of control and experimental rats at baseline and after 28 days of treatment with *Tetrapleura tetraptera* hydroethanolic extract and metformin

Groups	Days	HOMA-IR	HOMA-β
NCD	0	4.18(3.66—5.16)^bc^	200.98(143.36–346-67)^bc^
	28	4.23(3.71–4.77)^bc^	192.12(186.6–265.41)^bc^
	T28-T0	−0.10(−0.57–0.83)	22.96(−157.98–63.57)
HCHFD	0	12.31(10.78–14.96)^abc^	243.13(207.99–299.08)^bc^
	28	14.41(12.59–15.86)^abc^	274.65(230.55–355.33)^Sbc^
	T28-T0	1.8(0.9–2.9)	28.87(9.44–59.39)
HCHFD200	0	11.58(11–14.86)^abc^	238.48(222.92–284.91)^bc^
	28	5.31(4.85–6.41)^Sbc^	240.23(153.31–417.24)^bc^
	T28-T0	−6.36(−9.37_−5.38)	−5.57(−82.47–189.53)
DBC	0	28.24(21.39–29.41)^a^	53.34(44.03–89.46)^a^
	28	26.86(21.11–28.75)^a^	56.04(44.24–89.15)^a^
	T28-T0	−0.64(−3.01–0.13)	0.86(−0.31–3.52)
DB200	0	27.10(20.58–29.96)^a^	56.68 (49.59–83.27) ^a^
	28	8.74(6.82–10.73)^Sabc^	110.96(85.22–132.01) ^Sacb^
	T28-T0	−17.99(−19.22_−13.76)	49.23 (31.78–68.42)
DB400	0	26.63(21.14–32.28)^a^	54.44 (49.38–74.84)
	28	5.32(4.26–5.44)^Sbc^	142.75 (97.52–157.70) ^Sbc^
	T28-T0	−21.23(−28.01_−15.89)	79.00 (48.14–107.25)
DBMET	0	27.40(23.41–29.12)^a^	59.08 (49.53–66.67) ^a^
	28	6.48(5.4–7.28)^Sabc^	122.11 (93.53–185.55) ^Sbc^
	T28-T0	−21.22(−23.11_16.91)	70.99 (26.86–127.90)

^S^significant compared with T0 (p < 0.05). ^a^significant relative to normal control on the same treatment day(p < 0.05). ^b^significant compared with HCHFD on the same treatment day. ^c^significant compared with diabetic control on the same treatment day (p < 0.05). (n = 6)

### HET attenuated weight-gain in HCHFD rats

HCHFD rats gained more weight than NCD rats over the period of 4 weeks. Food intake and water intake were decreased in HCHFD rats compared with NCD rats; HET reduced food intake in HCHFD rats without changing significantly water intake. Energy intake was increased due to higher energy content of HCHFD diet. After 28 days of treatment, we noted that the weight gain was significantly lower in HCHFD treated rats than its untreated counterpart (p < 0.05) indicating that body mass increase was significantly suppressed in the HCHFD200 group compared with the HCHFD group. Of note, the dose-dependent weight loss that accompanied the diabetes status was greater in diabetic treated rats though not significant. Therefore we hypothesized that *T. tetraptera* could have a protective effect again obesity (Table 3).

**Table 3:** Body weight at baseline and after 28 days, food and water intakes of control and experimental rats treated with *Tetrapleura tetraptera* hydroethanolic extract and metformin

Groups	Weight T0 (g)	Weight T28 (g)	Weight T28-T0 (g)	Water intake (mL/day)	Food intake (g/day)
NCD	317(309–326)^bc^	332.5(320–336)^Sb^	13.5(4–26)	31.6(28.9–32.7)^b^	28.45(27.6–30.5)^bc^
HCHFD	414(388–425)^a^	452.5(445–468)^Sa^	43(20–63)	23.6(22.1–25.5)^a^	23.2(21.8–23.9)^ac^
HCHFD200	412(394–425)^a^	416(396–442)^ab^	6.5(−2–17)	21.45(20.6–24.1)^a^	17.55(16.8–19.4)^ab^
DBC	414(386–423)^a^	408(390–422)^a^	−2(−10–4)	22.45(20.6–23.9)^a^	16.7(15.6–17.5)^a^
DB200	413.5(390–426)^a^	410(388–421)^a^	−4(−8–−2)	21.2(20.4–23.4)^a^	17.6(16.4–18.6)^ab^
DB400	410.5(392–426)^a^	398(386–416)^a^	−7(−20–−2)	21.05(20.45–23.7)^a^	16.4(15.6–17.9)^ab^
DBMET	407(394–430)^a^	410(389–430)^a^	−2.5(−5–10)	23(21.4–24.7)^a^	17.6(16.9–19.5)^ab^

^S^significant compared with T0 (p < 0.05). ^a^significant relative to normal control on the same treatment day (p < 0.05). ^b^significant compared with HCHFD on the same treatment day. ^c^significant compared with diabetic control on the same treatment day (p < 0.05). (n = 6)

### HET possessed hypolipidemic effects and reduced tissue steatosis

Hyperlipidemia and related-tissue steatosis are among the most characteristic feature of T2DM and metabolic syndrome. These are also two major risk factors that contribute to the pathogenesis of cardiovascular diseases. Thus, to understand the effects of HET on lipid metabolism, the serum lipid profile and lipid accumulation in liver and skeletal muscle in T2DM rats were next investigated. As shown in Table 4, serum TG, total cholesterol, free fatty acids and LDL-cholesterol were considerably increased in both the HCHFD and HCHFD + STZ groups whereas HDL-cholesterol was reduced. The administration of HET (even at the dose of 200 mg/kg) reduced the serum level of TG, TC, FFA and increase that of the HDL-cholesterol. The effects of HET on TG, TC and FFA levels in livers and skeletal muscles from T2DM rats are presented in Table 5. A significant increase in liver and skeletal muscle FFA, TC and TG contents were observed in obese and T2DM rats and this effect was reversed near to the normal level by HET treatment (Table 5) in a dose-dependent manner (p < 0.05). Metformin had no significant effect on triglycerides and FFA in the serum and liver and HDL-cholesterol in the serum compared to the HCHFD, whereas there was a significant effect on total and LDL cholesterol.

**Table 4:** Total cholesterol (TC), triglycerides (TG), free fatty acids (FFA), low density lipoprotein cholesterol (LDL-C) and high density lipoprotein cholesterol (HDL-C) levels in the serum at the end of the study

Groups	TG (mg/dL)	TC (mg/dL)	HDL-C (mg/dL	LDL-C (mg/dL)	FFA (mg/dL)
NCD	86 (79–96)^bc^	79.5(78–87)^bc^	41.5(39–45)^bc^	21.9(16.6–26.8)^bc^	80(75–83)^bc^
HCHFD	173(164–183)^ac^	139.5(106–120)^ac^	24.5(19–26)^a^	79.8(78.6–89.2)^ac^	139.5(136–145)^ac^
HCHFD200	113(106–120)^abc^	109.5(106–120)^abc^	29.5(27–32)^abc^	57.2(54.6–67)^abc^	96.5(93–102)^abc^
DBC	205(187–220)^ab^	169(159–170)^ab^	20.5(17–23)^a^	106.6(98.8–112.6)^ab^	181(169–185)^a^
DB200	130(119–140)^abc^	119.5(116–123)^abc^	31.5(29–36)^abc^	61.3(57.2–69)^abc^	139(130–143)^ac^
DB400	90(86–93)^bc^	96.5(93–106)^abc^	43.5(40–45)^bc^	35.4(30.4–45)^abc^	80.5(74–86)^bc^
DBMET	195.5(176–205)^a^	129.5(125–137)^bc^	23.5(21–27)^a^	66.2(64.4–74.8)^abc^	146.5(136–150)^ac^

^a^significant relative to normal control (p < 0.05). ^b^significant compared with HCHFD (p < 0.05). ^c^significant compared with diabetic control (p < 0.05). (n = 6)

**Table 5:** Total cholesterol (TC), triglycerides (TG) and free fatty acids (FFA) levels in the liver and skeletal muscle at the end of the study

	Liver			Skeletal muscle		
Groups	TG (μg/mg)	TC (μg/mg)	FFA (μg/mg)	TG (μg/mg)	TC (μg/mg)	FFA (μg/mg)
NCD	80(76–83)^bc^	40.5(38–45)^bc^	17.5(15–21) ^bc^	26.50(25–32)^bc^	24(20–26)^bc^	4.87(4.5–5.36)^bc^
HCHFD	172(166–182)^a^	69(63–78)^a^	35(32–40)^ac^	45(41–48)^a^	39.5(38–42)^a^	8.16(7.69–8.98)^a^
HCHFD200	129(115–142)^abc^	50.5(47–53)^abc^	25.5(22–30)^bc^	32(28–36)^abc^	27(24–28)^bc^	6.09(5.68–6.4)^abc^
DBC	173.5(160–187)^a^	58.5(54–65)^ab^	43.5(36–48)^a^	44.5(39_-49)^a^	38.5(32–43)^a^	8.21(7.68–8.64)^a^
DB200	134(118–152)^abc^	48.5(45–52)^abc^	30(28–34)^ac^	29.5(25–33)^bc^	27.5(26–29)^bc^	6.1(5.7–6.5)^abc^
DB400	101(92–120)^abc^	43(39–46)^bc^	25.5(23–29)^bc^	27.5(23–31)^bc^	20.5(18–24)^bc^	4.62(4.1–5.8)^bc^
DBMET	163(150–184)^a^	41.5(38–45)^bc^	36.5(34–40)^a^	36.5(31–40)^abc^	22.5(20–26)^bc^	6.73(6.12–7.2)^abc^

^a^significant relative to normal control (p < 0.05). ^b^significant compared with HCHFD (p < 0.05). ^c^significant compared with diabetic control (p < 0.05). (n = 6)

### Besides exhibiting antioxidant ability, HET reduced glycated hemoglobin and AGE formation

Table 6 represents the levels of plasma HbA1C, MDA, GSH and SOD in all groups of rats at the end of this study. In obese and T2DM rats, significant increased levels of plasma HbA1C, uric acid and MDA and decreased levels of GSH and SOD were observed relative to the normal control. The alteration of these parameters was more pronounced in the diabetic group as MDA, HbA1C and uric acid levels in the DC rats were more than 50 % higher than in NC rats. The end product of lipid peroxidation, MDA, measured as thiobarbituric acid reactive substances (TBARS) revealed that obesity and type 2 diabetes enhanced lipid peroxidation, while HET, especially at a high dose, effectively attenuated lipid peroxidation and suppressed oxidative stress induced by HCHF diet and STZ, hence exhibiting good antioxidant capacity. The diabetic status promoted the Millard reaction and the generation of AGE as the amount of AGE exceeded 1.5-fold in the HCHFD + STZ group, compared with the normal control. However, administration of 400 mg/kg HET significantly lowered AGE generation by 35 % (Table 6) whereas the reduction of AGE formation by the lower dose was not significant. Unlike the diabetic group, the increase of glycated hemoglobin in the HCHFD was not accompanied by a significant increase in AGE.

**Table 6:** Plasma oxidative stress and antioxidant enzymes in treated and untreated high-carbohydrate high-fat fed and type 2 diabetic rats

Groups	TBARS (nmol/mg protein)	GSH (μmol/L)	Plasma uric acid (μmol/L)	SOD(Unit/mg protein)	HbA1C(g/kg Hb)	AGE (mg/mL)
NCD	5.33(4.98–6.20)^bc^	34(32–36)^bc^	31.8(28.9–34.1)^bc^	142(136–146)^bc^	55.5(50.8–60.1)^bc^	3.32(2.9–3.5)^c^
HCHFD	9.63(8.79–10.21)^a^	21(19–23)^a^	45.35(44.2–45.9)^c^	87(82–95) ^ac^	92.25(89.6–96.7)^c^	3.45(3.1–3.8)^c^
HCHFD200	6.92(6.52–8.20)^c^	27.5(25–29)^abc^	41.95(41.2–42.5)^abc^	137(129–144)^bc^	70.6(67.8–72.6)^abc^	3.4(2.9–3.7)^c^
DBC	10.44(9.57–11.20)^a^	22(19–24)^a^	56.65(54.8–58.3) ^ab^	74.5(72–80)^b^	103.8(99.3–108.3) ^ab^	5.05(4.6–5.3)
DB200	7.97(6.89–8.26)^abc^	29.5(27–32)^bc^	45.95(44.6–47.9) ^ac^	131.5(130–134)^bc^	78.55(76.3–81.5)^abc^	4.8(4.2–5.1)
DB400	5.94(4.6–7.39)^bc^	33.5(32–36)^bc^	33.75(32–35.4)^bc^	135(128–137)^bc^	51.45(49.3–55.2)^bc^	3.17(2.9–3.6)^c^
DBMET	6.22(4.8–7.36)^bc^	31.5(29–36)^bc^	36.3(35.8–37.3)^abc^	126(122–132)^abc^	61.05(57.6–62.3)^bc^	3.75(3.4–4.2)^c^

^a^significant relative to normal control (p < 0.05). ^b^significant compared with HCHFD (p < 0.05). ^c^significant compared with diabetic control (p < 0.05). (n = 6)

### HET improved liver and kidney functions

The hepatic injury was assessed by measuring serum alanine aminotransferase (ALT) and aspartate aminotransferase (AST) levels. Table 7 shows the levels of AST and ALT in plasma of normal control, obese rats, diabetic rats, and other treatment groups. The activities of these enzymes were found to be significantly (P < 0.05) higher in the serum of HCHFD and DBC groups than those in NCD group suggesting that HCHF and STZ independently caused hepatic injury in those animals. HET treatment also reversed the increase of serum ALT and AST in a dose-dependent manner.

**Table 7:** Plasma markers of hepatic and renal function in treated and untreated high carbohydrate high fat fed and type 2 diabetic rats

Groups	AST(IU/L)	ALT (IU/L)	Urea (mmol/L)	Creatinine (μmol/L)
NCD	122(119–126)^bc^	30.5(28–34)^bc^	5.65(4.60–6.2)	39.95(38.3–41.1)^bc^
HCHFD	150.5(146–154) ^ac^	56.5(54–59)^ac^	6.4(5.8–6.6)	47.1(46.2–49) ^a^
HCHFD200	132(129–136)^abc^	42(38–45)^abc^	5.75(5.5–6.2)	44.4(43.2–45.7) ^ac^
DBC	162(159–166) ^ab^	66(64–68)^ab^	6(5.6–7.4)	50.4(48.7–52.9) ^a^
DB200	135(133–138)^abc^	41.5(39–44)^abc^	5.7(4.3–6.5)	45.7(44.6–46.9)^ac^
DB400	122.5(120–126)^bc^	32.5(31–36)^bc^	5.65(5.1–5.9)	39.05(36.4–40.3)^bc^
DBMET	129(126–134)^bc^	39.5(36–42)^bc^	5.4(4.5–5.8)	42.4(41.8–44.6)^bc^

^a^significant relative to normal control (p < 0.05). ^b^significant compared with HCHFD (p < 0.05). ^c^significant compared with diabetic control (p < 0.05). (n = 6)

A significant increase in the level of creatinine were observed in type 2 diabetic rats whereas there were no significant changes in the urea levels of the animals, which were all in the normal range.

### HET improved inflammatory cytokines levels and cardiovascular function

To evaluate the effect of HET on inflammation and cardiovascular function in obese and type 2 diabetes rats, we next measured the levels of inflammatory cytokines, adiponectin, leptin and systolic blood pressure. Serum leptin concentrations were increased with HCHF diet groups compared with NCD whereas the concentrations of adiponectin were higher in the NCD (P < 0.05) when compared with the groups that received high-carbohydrate high-fat diet. Treatment with the higher dose of HET (400 mg/kg) and metformin increased the adiponectin and reduced leptin. Both adipocyte-derived hormones were not significantly affected by the lower dose of HET (Table 8). As depicted in Table 8 the serum levels of inflammatory cytokines, TNF- α and IL-6 and CRP levels were significantly increased. The administration of HET and metformin prevented the increase in TNF-α, IL-6 and CRP in serum in a concentration-dependent manner. Systolic blood pressure in HCHF diet–fed rats was increased as compared with NC diet–fed rats (Table 8). HET reduced systolic blood pressure in both HCHF200, DB200 and DB400 rats as compared with HCHF and DBC rats, respectively.

**Table 8:** Serum inflammatory markers, systolic blood pressure (SBP) and adiponectin in treated and untreated high-carbohydrate high-fat fed and type 2 diabetic rats

	NCD	HCHFD	HCHFD200	DBC	DB200	DB400	DBMET
CRP (μg/mL)	194.5(168–224)^bc^	621(589–652)^ac^	330.5(310–369)20.77^abc^	728.5(697–769)^ab^	350(329–375)^abc^	129.5(119–153)^abc^	328(299–356)^abc^
IL-6 (pg/mL)	155(139–169)^bc^	240.5(230–251) ^a^	182(175–192)^abc^	240(235–262)^a^	198.5(188–201)^abc^	150(143–160)^bc^	166(159–173)^bc^
TNF-α (μg/mL)	544.5(529–569)^bc^	851.5(816–873) ^a^	657.5(643–670)^abc^	878(847–900)^a^	605(582–623)^abc^	543(516–560)^bc^	595(579–610)^abc^
Leptin (ng/mL)	3.2(2.9–3.8)^bc^	5.5(4.6–5.9) ^ac^	3.93(3.66–4.36)^abc^	5.25(4.8–5.8)^a^	4.98(4.69–5.3)^b^	3.9(3.8–4.31)^bc^	3.12(2.7–3.26)^bc^
adiponectin (μg/mL)	45.85(42.9–47.1)^bc^	22.45(19.9–24.8) ^a^	32.25(29.7–33.6)^abc^	25.5(24.1–28.4)^a^	24.5(21.6–26.9)^a^	32.1(28.6–32.9)^abc^	29.65(28.4–32.1)^abc^
SBP (mmHg)	91(87–96)3.51^bc^	147.5(142–153) ^a^	113(110–117)^abc^	141(139–146)^a^	129.5(127–133)^abc^	108(101–112)^abc^	139.5(134–146)^a^

^a^significant relative to normal control (p < 0.05). ^b^significant compared with HCHFD (p < 0.05). ^c^significant compared with diabetic control (p < 0.05). (n = 6)

## Discussion

Type 2 diabetes, nonalcoholic fatty liver disease (NAFLD) and cardiovascular diseases are multiorgan dysfunction related to long-term metabolic syndrome, which remain major public health challenges in modern society [25]. Unregulated hyperglycemia, hyperlipidemia, oxidative stress, activation of polyol pathway and chronic low-grade inflammations, induced by sugars and lipids, delineate the combined sequence of metabolic derangements which may initiate changes in liver, kidneys, pancreas and cardiovascular structures and functions then, ultimately lead to cardiovascular disorders, nephropathy, neuropathy and retinopathy [26]. The risk factors which include central obesity, elevated blood pressure, inflammation, impaired glucose tolerance, insulin resistance, and dyslipidemia are also rsesponsible for the increased morbidity and mortality in humans. It is therefore, important to target these established biological alterations for the treatment and reduction of clustering risk factors of this syndrome. In this study we used a suitable animal model that mimics all these symptoms of human metabolic syndrome to test the potential pharmacological properties of *Tetrapleura tetraptera* in the management of obesity, diabetes, hypertension and related metabolic disorders. TT fruit pulp is a culinary spice which has long been used in traditional medicine to effectively treat diabetes and hypertension by local people in Ghana, Yoruba tribe of Nigeria as well as in southern and western part of Cameroon. Some researchers have demonstrated the anti-inflammatory and hyoglycemic properties of TT in a normal and T1DM Wistar rat model respectively [15]. Moreover, the hypotensive action of scopoletin, a coumarin isolated from the fruit of *T. tetraptera* was earlier reported more than three decade ago [27] in a study in which the intravenous administration of the compound at the dose 10–100 mg/kg reduced the arterial blood pressure of anaesthetized rats. Subsequent workers also demonstrated the hypotensive activities of the alcoholic extract of *T. tetraptera* fruits in cats, rats and rabbits [28]. However, the pattern of disease initiation and development in the models used in these studies was not closely analogous to the clinical situation of metabolic syndrome in humans, with characteristics of obesity, insulin resistance and hypertension, inflammation, hyperlipidemia and type 2 diabetes mellitus. In addition, this work focuses on the effects of this spice on high-fat high-fructose diet-induced weight gain and oxidative stress. In this study, HCHF diet fed obese rats and experimental T2DM rats induced by high-fat, high-carbohydrate diet and low-dose STZ presented with the symptoms as well as the associated complications of metabolic syndrome, including obesity, dyslipidemia, hypertension and impaired glucose tolerance followed by partial destruction of the β-cells of the pancreas. Thus, this produces a form of obesity and type 2 diabetes analogous to human form of the conditions than any other available animal model. The cardiovascular and hepatic complications included inflammation, steatosis along with increased plasma activities of transaminases. Indeed, besides presenting with insulin resistance syndrome as was characterized by the increased body weight, hypertriglyceridemia, hypercholesterolemia and compensatory hyperinsulinemia, HCHF fed rats showed mild hyperglycemia which was converted to frank hyperglycemia upon administration of low dose of STZ. As portrayed by the decrease in HOMA- β, this was accompanied by decline in secretory capacity of pancreatic beta cells to compensate for the existing insulin resistance. The concomitant decreased insulin level was still higher than that of the normal control. We demonstrated the effect of HET in lowering blood glucose and ameliorating insulin resistance. The HOMA-IR was reduced whereas HOMA-β was increased T2DM treated rats. This observation implied that HET could exert its hypoglycemic effect through improving peripheral IR and protecting pancreas islet β-cells and/or stimulating insulin secretion.

In addition to glucose abnormalities, T2DM patients are often likely to suffer from cardiovascular diseases as a consequence of dyslipidemia [29]. Because the insulin inhibits the hormone-sensitive lipase, IR may contribute to the development of lipid accumulation in hepatocyte through impairment of insulin’s capacity to subdue lipolysis. This event results in increase in the mobilization of FFA from the peripheral depots, elevated circulating FFA and development of hepatocyte steatosis [30]. In addition, skeletal muscle is another important insulin-responsive tissue besides liver, and a very strong relationship between IR in T2DM and both skeletal muscle steatosis and non-alcoholic fatty liver disease has been reported [31]. The HCHFD- induced intracellular accumulation of lipid metabolites in the liver and skeletal muscles further exacerbates insulin resistance through decreased tyrosine phosphorylation of insulin receptor substrate, a key mediator in insulin action [32]. Both insulin deprivation and insulin resistance attenuate the activity of lipoprotein lipase, a key enzyme in the removal and degradation of triglycerides from circulation [33]. This leads to hypertriglyceridemia as a result of both increased hepatic VLDL overproduction and impaired catabolism of triglyceride rich particles. Metabolic disorders such as dyslipidemia are observed in the majority of T2DM patients and represents an independent risk factor for the development of coronary heart disease in people with type 2 diabetes [34]. Likewise, lipid accumulation in nonadipose tissues could lead to cell dysfunction and/or cell apoptosis [35]. Previous research demonstrated that increased hepatocyte apoptosis is a hallmark of NAFLD in obese individuals [36]. In this study, we observed a significant elevation of TG, TC and FFA levels in serums, livers, and skeletal muscles of HCHFD obese and T2DM rats. On the other hand, level of HDL-C in serum was downregulated in obese and T2DM animals. HET exhibited a strong activity in the modulation of lipid metabolism and alleviated these abnormalities in a dose dependent way. Interestingly, the effect of HET at the dose 200 mg/kg was greater than that of metformin (300 mg/kg) in lowering TG and FFA in serum and organs, which in addition to its hypoglycemic effect is potential to protect patients with metabolic syndrome from cardiovascular disease.

High-carbohydrate, high-fat diet–induced oxidative stress has been linked to the initiation of symptoms of MetS. During the pathogenesis of T2DM, chronic hyperglycemia results in increased production of reactive oxygen species (ROS) and appears to constitute the key and common events in the T2DM [37]. Persistent oxidative stress may further contribute to the vicious cycle of IR, in T2DM patients with concomitant increased lipid peroxidation as analyzed with TBARS, and altered antioxidant defense [38]. It is reported that increased level of plasma MDA, an oxidative stress marker, and the reduced activities of endogenous antioxidant enzyme SOD and the level of GSH have been found in T2DM patients [39]. This observation is consistent with our results, which showed that in obese and T2DM rats, plasma levels of GSH and SOD were reduced and that of MDA was increased as compared to healthy rats. Oral administration of HET significantly reduced plasma MDA level and increased levels of GSH and SOD in a dose-dependent manner in obese and T2DM rats as compared to vehicle treatment. In the plasma of the diabetic animals we also observed an elevation of advanced glycation end products, and HbA1C, an early product of glycation process, that accompanied lipid peroxidation. In addition to its antioxidant effect, HET suppressed the formation of glycated hemoglobin and AGE, which could be pathogenic biomarkers in type 2 diabetes-associated vasculopathy. Liver and kidneys are organs of metabolism and excretion, and are constantly endowed with the task of detoxification of environmental pollutants, xenobiotics and chemotherapeutic agents. AST, ALT, are the major hepatic marker enzymes whereas urea and creatinine are markers of renal function. In the current study, leakage from damaged cells results in the elevation of hepatic markers in the plasma, thus, reflected the hepatocyte damage. In another aspect the significant increase of the creatinine and the non-significant elevation of urea observed in HCHFD and type 2 diabetic rats indicated modifications in protein catabolism, deleterious effects of free radicals and alterations in renal function during diabetes. Treatment with HET significantly decreased the altered levels of blood AST, ALT, and creatinine.

We also observed an increase in uric acid, likely due to the sucrose feeding. In fact, fructose is a component of sucrose and is distinct from other sugars in its ability to cause hepatic intracellular ATP depletion (which stimulates food intake), nucleotide turnover, and the generation of uric acid [40]. Recent studies showed that fructose-induced uric acid generation causes mitochondrial oxidative stress that alters metabolism and stimulates visceral fat accumulation independent of excessive energy intake [41]. Epidemiological studies have also linked fructose intake with hypertension and elevated fasting uri acid levels [42]. It has been also reported that long-term feeding of high fructose containing diet causes nonalcoholic fatty liver disease and hyperlipidemia which is directly linked to the development of insulin resistance, the first major pathogenesis of T2DM [40]. This accounts for why fructose intake increases the risk for metabolic syndrome. Indeed, the intake of higher amounts of fructose particularly from added sugars, such as table sugar (sucrose), simple carbohydrates, soft drinks and high-fructose corn syrup has increased dramatically in the last century and is largely associated with the rise in overweight, obesity, followed by insulin resistance, diabetes, and metabolic syndrome in Americans [43]. However, the mechanism by which fructose increases weight is likely via its ability to stimulate hunger, block satiety responses, and reduction in resting energy expenditure in overweight and obese subjects [44]. Henceforth, weight gain observed in this study was driven primarily by increased energy intake from fat and reduced metabolism rate as a consequence of high fructose intake induced leptin resistance in rats [44]. With regard to the adipocytokines levels, adiponectin and leptin secreted mainly by adipose tissue are present in adipocytes and serum [3, 45]. Serum levels of leptin are strongly associated with fat mass, BMI and leptin resistance in obesity and also tend to increase in diabetes, hypertension, hyperlipidemia, and ischemic heart disease [3, 45]. This was consistently observed in our study where leptin levels clearly increased whereas adiponectin decreased in the HCHFD groups compared with the NCD groups. Besides, leptin is also involved in the regulation of energy balance and production of inflammatory cytokines [46].

Chronic low-grade inflammation is believed to play an important pathogenetic role in the development of IR and T2DM and it is therefore closely related to reduced insulin sensitivity [47]. C-reactive protein,, an acute-phase protein, is an inflammatory marker synthesized and released by the liver under the stimulation of cytokines such as TNF- α and IL-6 [48]. Earlier studies have demonstrated that CRP, TNF-α and IL-6, circulating markers of low-grade inflammation and vascular injury, are strong predictors of increased risk for T2DM [49]. On the other hand, adiponectin is inversely correlated with obesity and has a protective effect against insulin resistance as it exhibits anti-inflammatory properties by reducing TNF-α and IL-6 secretion from macrophages [50]. Our results indicated that significant increases in the levels of pro-inflammatory cytokines, TNF- α IL-6 and also CRP were observed in obese and type 2 diabetic rats and were consistent with other studies [3, 5, 6, 45, 51]. Upon administration of HET, we observed a significant reduction in serum cytokine levels and CRP associated with a decrease in insulin resistance.

Another leading risk factor for cardiovascular and renal morbidity and mortality is hypertension. It is also one of the major contributors to global disease burden (4.5 %) and is prevalent in both developing countries and the developed world [52]. In obesity, adipocytes also expressed angiotensinogen, leading to an angiotensin II–induced increase in blood pressure [53]. Besides reducing weight gain, the hypotensive effect of HET in obese and type2 diabetic rats observed in this study, corroborated earlier reports by other abovementioned researchers.

Studies have found that *T. tetraptera* fruit is a reservoir of medicinal phytochemicals with wide range of potential application [21, 17]. For instance, it has been reported that the pulp of dried fruits contains about 1 to 20 % of saponins, 2.5 % of flavonoids, 0.12 % of tannins, 1 % alkaloids 5 % phytates, and 5–11.36 % of fibers [17], among which flavanoids and saponins, fibers, and at a lesser extent phytates are well known for at least one of the following antidiabetic, anti-inflammatory, antioxidant and anti-obesity properties [5, 7, 51]. Of note, Fleischer et al. [54] have recently isolated three flavonoids from the fruits of *Tetrapleura tetraptera*, which were the first reported isolation of such constituents from the genus Tetrapleura. In addition, the isolated flavonoids have demonstrated various biological activities. For Instance, 2’, 4’, 4-tetrahydroxychalcone (butein) has been shown to be a potent antioxidant and an anti-inflammatory agent [55]. Secondly, 2’,4,4’-trihy-droxychacone (Isoliquiritigenin) has exhibited vasorelaxant effect on the phenylephrine-precontracted rat aorta [56], aldose reductase inhibiting property [57] and is also a potent anti-tumor and anti-inflammatory agent [58]. The third isolated compound 4’, 5, 7-trihydroxy-flavanone (Naringenin) has been reported to lower plasma cholesterol in vivo [59] and also has antioxidant and blood glucose lowering activities [60]. Therefore the presence of these constituents in the fruit of *T. Tetraptera* could have contributed to the observed antioxidant, anti-inflammatory, hypolipidemic, hypoglycaemic and hypotensive effects of the extract in our study.

In conclusion, the results of this study provides the scientific rationale for some of the numerous uses of *Tetrapleura tetraptera* fruit as in the management of type 2 diabetes, inflammation and cardiovascular diseases like hypertension and stroke in some folkloric societies of Cameroon, Nigeria and Ghana. Besides, it has a protective effect against diet-induced obesity.
